# Acute Myocardial Infarction in Young Systemic Lupus Erythematosus Patient with Normal Coronary Arteries

**DOI:** 10.7759/cureus.1370

**Published:** 2017-06-19

**Authors:** Ali Farooq, Aman Ullah, Farman Ali, Hassaan Yasin, Waseem Amjad, Muhammad Pervaiz

**Affiliations:** 1 Internal Medicine, West Virginia University - Charleston Division; 2 Internal Medicine, St Joseph Mercy Oakland Hospital; 3 Medicine, St.john Hospital and Medical Center, Detroit; 4 Forest Hills Hospital, Northshore-Long Island Jewish Health System; 5 Cardiology, West Virginia University - Charleston Division

**Keywords:** systemic lupus erythematosus, acute myocardial infarction, normal coronary arteries

## Abstract

A 34-year-old female with a past medical history of systemic lupus erythematosus (SLE) and a deep venous thrombosis experienced substernal chest pain for 24 hours. Her physical exam was remarkable for brown macular rash over the face. Her initial electrocardiogram showed ST depression in lead V3–V6 along with an elevated troponin I level of 1.23 ng/dl (normal 0.0–0.4) that increased to 2.33 ng/dl in a four-hour duration. Cardiac catheterization revealed mild 10–20% focal plaque in the mid left anterior descending artery and otherwise normal coronary arteries. Laboratory data revealed an erythrocyte sedimentation rate of 98 mm/hour (normal 1–20), C-reactive protein of 25 mg/L (normal 0.0–2.9), and positive antinuclear antibody. In the absence of a significant coronary atherosclerosis along with elevated inflammatory markers, inflammation of coronary microcirculation was considered as an underlying pathophysiology of myocardial infarction. The patient was started on immunosuppression therapy with hydroxychloroquine and prednisone. Her chest pain improved and she was discharged in a stable condition. The patient remained stable and symptom-free over a follow-up period of nine months.

## Introduction

Ischemic heart disease is a common complication of systemic lupus erythematosus (SLE) and may be observed in up to 16% of SLE patients [1]. The most common underlying etiologies of acute myocardial infarction (AMI) in SLE patients include coronary atherosclerosis, thrombosis, arteritis, and myocarditis. The role of coronary microvascular inflammation in cardiovascular complications is well established in vasculitic syndromes [2-3]. Inflammatory lesions of the coronary microcirculation are less commonly recognized as an etiology of AMI in SLE. We report a case of AMI in an SLE patient with normal coronary arteries in whom coronary microcirculation vasculitis was considered as the origin of AMI and as a result of immunosuppressive therapy, her angina improved.

## Case presentation

**Figure 1:**
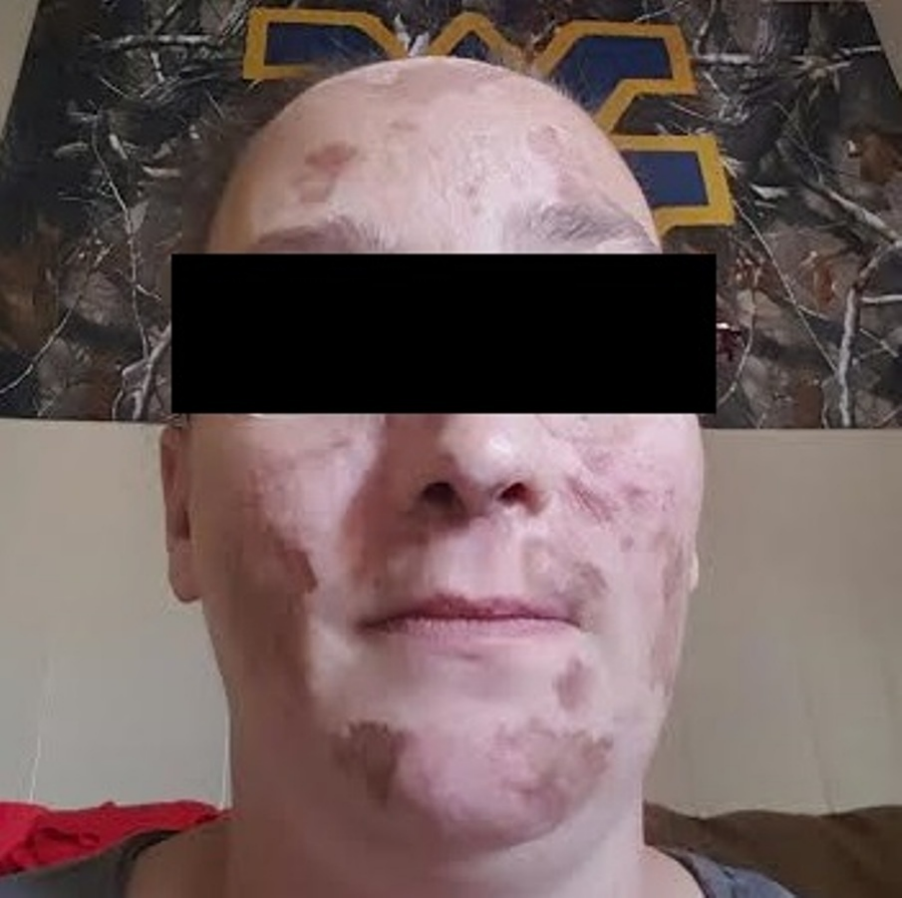
Diffuse brown macular rash over the face

A 34-year-old female with a history of SLE and a deep venous thrombosis presented to the hospital with substernal chest pain that had persisted for 24 hours. She had a history of smoking one pack/day for five years and no other cardiovascular risk factors. Her only home medications were apixaban 5 mg twice a day and clobetasol topical wash. The patient was started on azathioprine for SLE by her rheumatologist, but she missed her recent appointment with the rheumatologist and did not fill the azathioprine prescription for about six months. A physical examination revealed a blood pressure of 120/70, a heart rate of 78 beats/minute, a respiratory rate of 18 cycles/minute, and a temperature of 38 °C. Her dermatological examination was remarkable for brown macular rash over the face (Figure 1).

**Figure 2:**
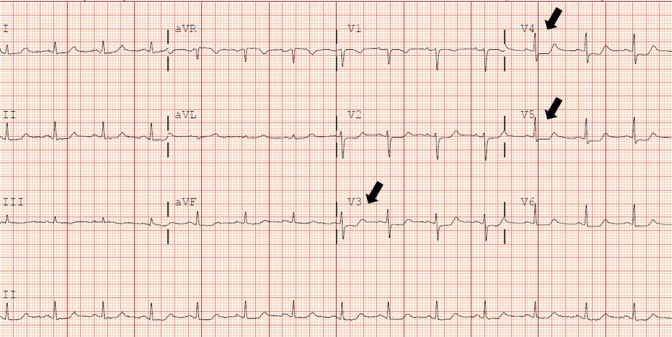
ST depression in lead V3–V5

The lungs were clear to auscultation bilaterally and the heart sounds were normal; no rub, gallop, or murmurs were present. An electrocardiogram revealed ST depression in lead V3–V6 (Figure 2).

**Figure 3:**
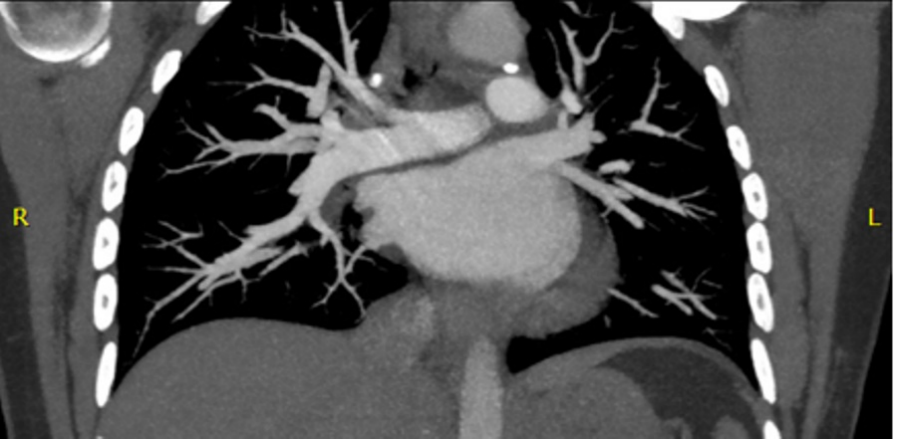
Computed tomography angiogram of pulmonary arteries showing no pulmonary embolus

The troponin I level, which was 1.23 ng/dl (normal 0.0–0.04) at presentation, increased to 2.33 ng/dl in a four-hour period. A chest computed tomography with contrast obtained in the emergency department did not show pulmonary embolism (Figure 3).

Her other laboratory work revealed elevated inflammatory markers. The erythrocyte sedimentation rate was 98 mm/hour (normal 1–20), C-reactive protein was 25 mg/L (normal 0.0–2.9), and antinuclear antibody was positive. A skin biopsy of the left superior eyebrow revealed chronic interface dermatitis and vasculitis.

**Figure 4:**
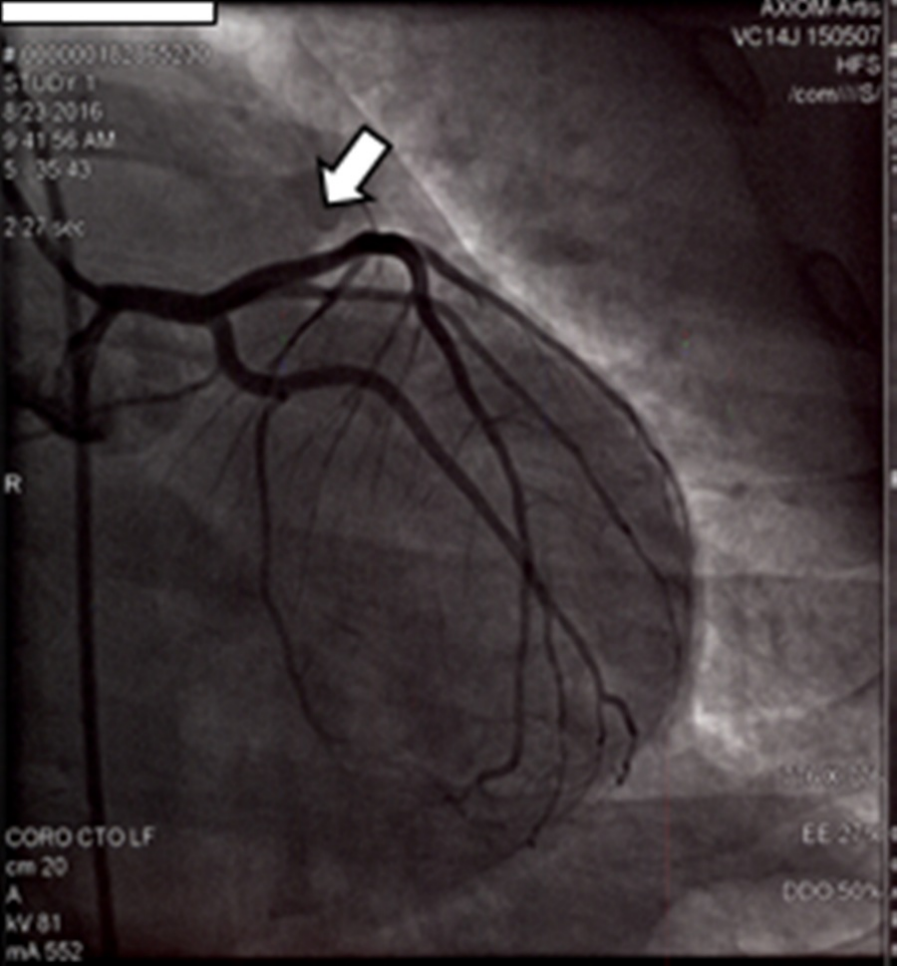
Coronary angiography revealed a mild 10–20% mid focal stenosis of the mid left anterior descending artery

A coronary angiography that was performed revealed a mild, single vessel coronary artery disease with 10–20% focal plaque in the mid left anterior descending artery. There were no signs of coronary arteritis, e.g., coronary aneurysm or dissection (Figure 4).

An echocardiogram revealed a normal left ventricular ejection fraction of 60–65%. Cardiac magnetic resonance (CMR) imaging revealed normal ventricular wall thickness; no hyperemia, edema or wall motion abnormalities were detected.

Normal coronary arteries on angiography, active vasculitis of discoid rash on biopsy, and elevated inflammatory marker protein supported the idea of coronary microcirculation vasculitis as the etiology of AMI.

The patient was started on aspirin 81 mg/day, atorvastatin 40 mg/day, hydroxychloroquine 200 mg/day, and prednisone 30 mg/day, and she was continued with apixaban. Her chest pain improved and she remained complication free during hospitalization. The patient was continued on the same medication at discharge and the prednisone was tapered off after one month. A repeat echocardiogram after a six-month period revealed a normal left ventricular ejection fraction of 60–65%, and no ventricular wall motion abnormalities or thickness. The patient has been followed closely over a period of nine months and has remained symptom-free and stable without complications.

## Discussion

Accelerated coronary atherosclerosis is a frequent cause of AMI in SLE due to the association of SLE with risk factors for coronary atherosclerosis or because of prolonged steroid therapy [4]. In the absence of coronary atherosclerosis on coronary angiography, the pathophysiology of myocardial infarction can be explained on the basis of coronary arteritis, coronary artery thrombosis or embolization with spontaneous recanalization, and myocarditis [5]. Coronary arteritis appears as coronary aneurysm or dissection on angiography. There was no evidence of coronary arteritis on the angiogram in our patient. CMR and tissue Doppler echocardiographic imaging are exquisitely sensitive to myocardial contractile function. There were no signs of myocarditis on CMR or echocardiogram in our patient. In the absence of atherosclerosis on coronary angiogram, small vessel vasculitis was considered as the origin of AMI. Elevated inflammatory markers along with active vasculitic skin lesions on the face also contributed toward the diagnosis. Improvement of the anginal symptoms after starting the patient on immunosuppressive therapy with hydroxychloroquine and prednisone also favors coronary microcirculation vasculitis as the origin of AMI. Although coronary angiogram is the gold standard test for detecting coronary atherosclerosis, it is not helpful in visualizing small vessels where the disease may lie [6]. In this scenario, endomyocardial biopsy to confirm the diagnosis of vasculitis is infrequently performed because of the overall reported rate of complications of up to 6% [7].

## Conclusions

In SLE patients presenting with AMI, especially in the absence of the most common causes of AMI, coronary atherosclerosis, arteritis, myocarditis, one should consider small vessel vasculitis as an etiology of AMI. Immunosuppressant therapy to treat vasculitis is a plausible treatment option. However, there is a need for more evidence-based studies to ascertain the natural disease course in this condition because starting young patients on immunosuppressant therapy places this patient population at risk of adverse effects.
